# Initial shockable rhythm detection among patients with out-of-hospital cardiac arrest by arrest location: A population-based cohort study in Japan

**DOI:** 10.1016/j.resplu.2026.101542

**Published:** 2026-10-29

**Authors:** Chika Nishiyama, Tomonari Shimamoto, Kosuke Kiyohara, Tetsuhisa Kitamura, Katsuya Tanaka, Tetsuya Sakamoto, Taku Iwami

**Affiliations:** a Department of Critical Care Nursing, Human Health Sciences, Graduate School of Medicine, Kyoto University, Kyoto, Japan; b Department of Preventive Services, Graduate School of Medicine, Kyoto University, Kyoto, Japan; c Department of Food Science, Faculty of Home Economics, Otsuma Women’s University, Tokyo, Japan; d Division of Environmental Medicine and Population Sciences, Department of Social and Environmental Medicine, Graduate School of Medicine, Osaka University, Osaka, Japan; e Toyonaka City Fire Department, Toyonaka, Japan; f Department of Emergency Medicine, Teikyo University School of Medicine, Tokyo, Japan

**Keywords:** Out-of-hospital cardiac arrest (OHCA), Ventricular fibrillation (VF), Automated external defibrillators (AED), Population-based cohort

## Abstract

**Background:**

We described the patient characteristics, bystander interventions, and outcomes after out-of-hospital cardiac arrest (OHCA) according to arrest location, focusing on initial rhythm and automated external defibrillator (AED) use.

**Methods:**

We analyzed medical-origin OHCAs from a population-based registry (2016–2024) by arrest location: home, nursing home, or other locations. Patient characteristics, bystander interventions, and outcomes were summarized descriptively. Multivariable logistic regression examined the association between arrest location and initial shockable rhythm.

**Results:**

Among 2478 OHCA cases, initial shockable rhythm was identified in 36.3% (221/609) in nursing homes, 7.1% (113/1582) at home, and 33.4% (96/287) in other locations. After adjustment, the odds of an initial shockable rhythm were higher in nursing homes than at home (adjusted OR, 12.35; 95% CI, 8.93–17.24) and than in other locations (adjusted OR, 2.35; 95% CI, 1.60–3.45). In nursing homes, 96.8% (214/221) of patients with an initial shockable rhythm received an AED shock. One-month survival with favorable neurological outcomes was the lowest in nursing homes (0.8% [5/609]).

**Conclusions:**

Initial shockable rhythm was identified in more than one-third of arrests occurring in nursing homes, and 97% of these patients received an AED shock. This finding may partly reflect earlier AED access, potentially increasing the likelihood of detecting a shockable rhythm soon after collapse. These findings underscore the importance of early rhythm detection through prompt AED use and suggest that extending AED access to home settings warrants further investigation.

## Highlights

• Initial shockable rhythm was observed in over one-third of OHCAs in nursing homes.
• 97% of initial shockable-rhythm cases in nursing homes received an AED shock.
• Earlier AED application may facilitate detection of an initial shockable rhythm.

## Introduction

Out-of-hospital cardiac arrest (OHCA) is a major public health burden worldwide.^1^ Despite efforts to improve OHCA outcomes, mortality remains high.^2^ Early bystander cardiopulmonary resuscitation (CPR) and early defibrillation using automated external defibrillators (AEDs) are critical for survival after OHCA.

Ventricular fibrillation (VF) often deteriorates into a non-shockable rhythm within minutes after collapse; therefore, delayed AED application may miss the opportunity for defibrillation.^3^ Previous studies have shown that the location of cardiac arrest strongly influences both access to AEDs and subsequent outcomes.^4^, ^5^ Cardiac arrests occurring at home are less likely to be witnessed and to present with shockable rhythms, resulting in poor outcomes.^5^, ^6^

Japan is among the world’s most rapidly aging societies,^7^ with a median age of OHCA patients of approximately 80 years.^8^ The proportion of individuals residing in nursing homes has steadily increased, making these facilities an increasingly common setting for OHCA. Although OHCA patients in nursing homes may have characteristics similar to those of patients at home, AEDs are often readily available in these facilities, potentially enabling earlier rhythm analysis and detection of VF after collapse. The aim of this study was to describe the patient characteristics, bystander interventions, and outcomes after OHCA according to arrest location, with a particular focus on initial rhythm and AED use.

## Methods

### Study design and setting

This descriptive study used a prospective population-based cohort conducted in Toyonaka City, Osaka, Japan, from 2016 to 2024. Japanese emergency medical service (EMS) personnel are required to initiate resuscitation and transport all OHCA patients to a medical institution if they were called, even when a do not attempt resuscitation (DNAR) order is present, unless obvious signs of death were observed. EMS personnel were not permitted to terminate resuscitation in the prehospital setting. Details of the EMS in Toyonaka City have been reported previously.^9^, ^10^, ^11^

### Data collection

EMS personnel collected data on all OHCA patients for whom resuscitation was attempted according to the Utstein-style guidelines,^12^ including sex, age, arrest location, activities of daily living (ADL) before arrest, categorized as good, moderate disability but independent, or severe disability requiring assistance, as assessed by EMS personnel based on information obtained at the scene, first documented cardiac rhythm, bystander-initiated CPR, AED shock, resuscitation time course, prehospital return of spontaneous circulation, 1-month survival, and neurological status at 1 month. Additionally, EMS personnel assessed the quality of bystander CPR by directly observing its performance in the field; high-quality bystander CPR required correct hand position, a compression rate of 100–120/min, and a compression depth of ≥5 cm. Methods and definitions have been described previously.^9^

### Statistical analysis

Patients of all ages with OHCA of medical origin for whom resuscitation was attempted by EMS personnel were included. Patients with EMS-witnessed or unknown locations were excluded. Cases were categorized into three location groups: home, nursing home, and other locations. Other locations included roads, workplaces, schools, public facilities, sports facilities, railway stations, airports, and other miscellaneous locations. Based on the Utstein definition,^12^ initial shockable rhythm was defined as ventricular fibrillation (VF) or pulseless ventricular tachycardia (VT) on the first EMS-recorded rhythm, or as a rhythm classified as shockable by an AED. Descriptive statistics were performed, with continuous variables reported as medians and interquartile ranges (IQR) and categorical variables as counts and percentages. Exact 95% confidence intervals (CIs) were calculated using the Clopper–Pearson method. To examine the association between arrest location and initial shockable rhythm while accounting for differences in patient and arrest characteristics, a multivariable logistic regression analysis was performed with initial shockable rhythm as the dependent variable. Arrest location was included as the main explanatory variable, with adjustment for age, sex, pre-arrest ADL, and witnessed status. Adjusted odds ratios (ORs) and 95% CIs were calculated. All statistical procedures were performed using IBM SPSS version 27.0 J (IBM Corp, Armonk, NY, USA).

### Ethics

The study was conducted according to the Declaration of Helsinki and the Ethical Guidelines for Medical and Health Research Involving Human Subjects of Japan. The study protocol was approved by the Ethics Committee of Kyoto University (R2790-2). The data were anonymized at the Toyonaka City Fire Department, and informed consent was waived.

## Results

Between January 2016 and December 2024, 3270 OHCA cases were identified. Among the enrolled patients, those who did not receive resuscitation attempts by EMS personnel (n = 79), those whose cardiac arrest was witnessed by EMS personnel (n = 214), those with a non-medical origin (n = 494), those with missing data on etiology (n = 3), and those without data regarding the location of arrest (n = 2) were excluded. Finally, 2478 patients were included in the analysis: 1582 at home, 609 in nursing homes, and 287 in other locations (Fig. 1).

**Fig. 1:**
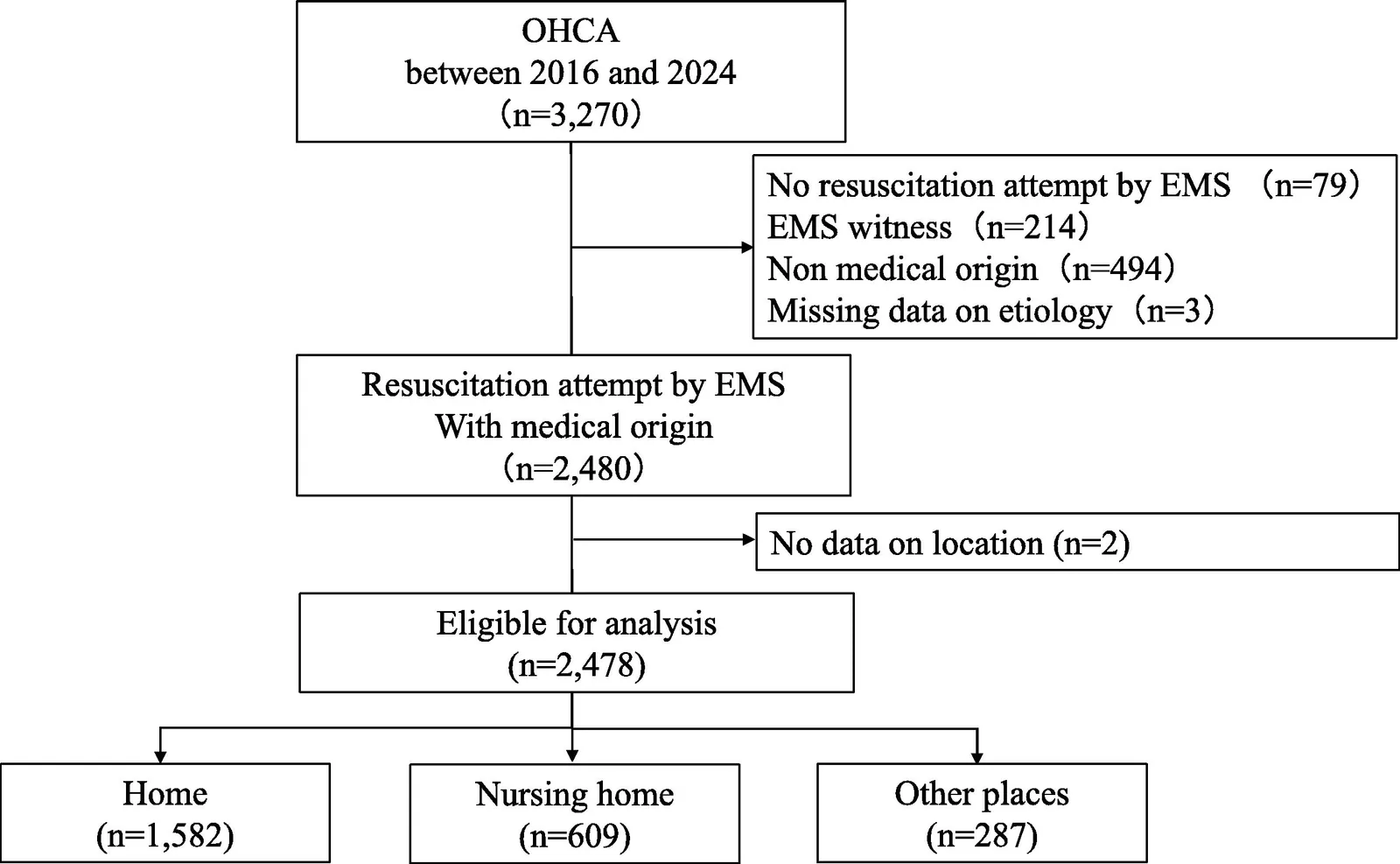
**Patients flow**. OHCA denoted out-of-hospital cardiac arrest; EMS, emergency medical services.

Table 1 summarizes patient characteristics, bystander interventions, and outcomes by arrest location. Of these, 63.8% occurred at home, 24.6% at nursing homes, and 11.6% at other locations. Patients in nursing homes were older (median 88 years [IQR 82–92]) and were less likely to have good pre-arrest ADL (11.7%) compared with those at home (80 years [72–86], 60.4%) and in other locations (69 years [56–79], 64.8%). Initial shockable rhythm was identified in more than one-third of arrests occurring in nursing homes (36.3%), compared with 7.1% at home and 33.4% in other locations. In nursing homes, AED shocks were delivered in 96.8% (214/221) of initial shockable rhythm cases, compared with 1.8% (2/113) at home and 38.5% (37/96) in other locations. After adjustment for age, sex, pre-arrest ADL, and witnessed status, the adjusted odds of initial shockable rhythm were higher in nursing homes than at home (adjusted OR, 12.35; 95% CI, 8.93–17.24) and than in other locations (adjusted OR, 2.35; 95% CI, 1.60–3.45). The proportion of high-quality CPR was highest in other locations (77.6%), followed by nursing homes (52.5%). One-month survival with favorable neurological outcomes was lowest in nursing homes (0.8%) and highest in other locations (12.9%).

**Table 1:** Patient characteristics, bystander interventions, and outcomes by arrest locations.

	**Home**	**Nursing home**	**Other locations**
	**n = 1582**	**n = 609**	**n = 287**
**Patient characteristics**			
Sex, male, n (%)	962 (60.8)	209 (34.3)	204 (71.1)
Age, years, median (IQR)	80 (72–86)	88 (82–92)	69 (56–79)
**Age category, n (%)**			
0–17 years	24 (1.5)	0	0
18–64 years	198 (12.5)	10 (1.6)	111 (38.7)
65–74 years	290 (18.3)	33 (5.4)	71 (24.7)
≥75 years	1070 (67.6)	566 (92.9)	105 (36.6)
Pre-arrest ADL, good, n (%)	956 (60.4)	71 (11.7)	186 (64.8)
Witnessed arrest, n (%)	578 (36.5)	230 (37.8)	164 (57.1)
Initial shockable rhythm*, n (%) [95% CI]	113 (7.1) [5.9–8.5]	221 (36.3) [32.5–40.3]	96 (33.4) [28.0–39.2]
**Bystander interventions**			
Dispatcher-assisted CPR instruction, n (%)	1160 (73.3)	438 (71.9)	147 (51.2)
Bystander CPR, n (%)	710 (44.9)	486 (79.8)	143 (49.8)
Provision of high-quality CPR by bystander**, n (%)	303 (42.7)	255 (52.5)	111 (77.6)
AED shock delivered, n (%) [95% CI]	2 (0.1) [0.02–0.5]	214 (35.1) [31.3–39.1]	37 (12.9) [9.2–17.3]
**EMS resuscitation time intervals**			
Call-to-scene arrival, minutes, median (IQR)	6 (5–7)	6 (4–7)	6 (5–7)
Call-to-hospital arrival, minutes, median (IQR)	35 (30–40)	35 (30–41)	32 (27–38)
**Patient outcomes**			
ROSC before hospital arrival, n (%)	212 (13.4)	68 (11.2)	72 (25.1)
1-month survival, n (%)	95 (6.0)	16 (2.6)	65 (22.6)
1-month survival with favorable neurological outcome, n (%) [95% CI]	43 (2.7) [2.0–3.6]	5 (0.8) [0.3–1.9]	37 (12.9) [9.2–17.3]

IQR denotes interquartile range, ADL; activities of daily living, AED; automated external defibrillator, CI: confidence interval, CPR; cardiopulmonary resuscitation, EMS; emergency medical service, ROSC; return of spontaneous circulation, VF; ventricular fibrillation.

* VF or pulseless VT on the first EMS-recorded rhythm, or as a rhythm classified as shockable rhythm by an AED.

** Percentages are calculated among patients in which bystander CPR was performed. (n = 710 at home, n = 486 at nursing home, n = 143 at other locations).

## Discussion

In this study, we analyzed data from 2478 OHCA patients to describe patient characteristics, bystander interventions, and outcomes according to arrest location, focusing on initial shockable rhythm and AED use. Notably, initial shockable rhythm was identified in more than one-third (36%) of arrests occurring in nursing homes, and 97.0% of these cases received an AED shock.

The proportion of initial shockable rhythm in nursing homes was markedly higher than that reported in previous studies.^13^, ^14^, ^15^ After adjustment, the odds of an initial shockable rhythm remained substantially higher in nursing homes than at home and in other locations. One possible explanation for this difference is greater AED availability and use before EMS arrival. Public-access AEDs have been widely disseminated throughout Japan,^16^ with medical or nursing facilities reported as one of the major types of locations where public-access AEDs are deployed.^10^ In addition, a population-based study in Tokyo reported more frequent AED shock delivery in nursing homes than in private residences.^17^ In our study, 97% with an initial shockable rhythm in nursing homes received an AED shock before EMS arrival. In contrast, none of the nursing-home patients in the Australian study received a bystander AED shock,^13^ and valid data on AED availability or use were unavailable in the Swedish study.^14^ In the English study, which used the same definition of shockable rhythm as ours, AED availability data were missing in 63.2% of cases, and an AED was applied by a bystander in only 8.8% of cases.^15^ Although the published data do not indicate how many shockable rhythms were identified by an AED before EMS arrival, the low proportion of bystander AED application suggests that this may have occurred in only a limited proportion of cases. Thus, differences in AED availability and use before EMS arrival may partly account for the markedly higher proportion of initial shockable rhythm observed in our study. These findings raise the possibility that, even in home settings where shockable rhythms have traditionally been considered uncommon, earlier AED application may increase the likelihood of identifying a shockable rhythm before it deteriorates to a non-shockable rhythm. However, AED availability and the timing of AED application were not directly assessed in our study. Therefore, a causal relationship between earlier AED use and the higher proportion of initial shockable rhythm cannot be established, and differences in study populations or EMS systems may also have contributed to the observed variation.

The clinical benefit of early defibrillation in nursing-home residents may be limited by their advanced age and poor pre-arrest functional status, which may reflect reduced physiological reserve. Nevertheless, our findings in nursing homes may also have implications for AED use in non-nursing-home settings. Patients at home and other locations were younger and had better pre-arrest ADL than those in nursing homes, suggesting that early AED access may be relevant in these settings. The effectiveness of AED use for OHCA occurring at home has been questioned.^18^ Patients with OHCA at home are less likely to present with shockable rhythm and tend to have worse outcomes than patients in public locations.^19^, ^20^ A recent study supported the clinical effectiveness of AEDs for shockable rhythms occurring at home, but also indicated that routine household purchase of AEDs might not be cost-effective at current pricing.^21^ However, both the effectiveness and cost-effectiveness of AED deployment critically depend on the ability to identify shockable rhythm and deliver timely defibrillation shocks. In our study, only 36.5% of cases were witnessed by a bystander, suggesting a need to improve early recognition of cardiac arrest at home. Given that approximately 70–80% of OHCAs occur at home, strategies targeting the home settings warrants further investigation. Future approaches may need to extend beyond AED placement alone and include: (1) leveraging digital technologies to improve early recognition of collapse and earlier activation of the emergency response system, including AED retrieval; (2) implementing systems that dispatch nearby bystanders to the scene of cardiac arrest and direct them to the nearest AED to facilitate early defibrillation; and (3) utilizing wearable devices and artificial intelligence to identify individuals at high risk of sudden cardiac arrest.^1^

This study has several limitations. First, although we had data on whether an AED shock was delivered, we did not have information on whether AED pads were applied or whether an AED was available at the location of arrest; therefore, we were unable to determine the proportion of cases in which an AED was applied. Second, we were unable to obtain information about several factors associated with the occurrence and/or prognosis of cardiac arrests, including past medical history, medications, and lifestyle factors, comorbidity burden, and frailty before the arrest. Third, data on DNAR orders were not available in our registry, which may have influenced patient selection for resuscitation and the interpretation of outcomes. Fourth, as with other registry-based studies, misclassification of recorded variables may have occurred, and missing data may have introduced bias. Fifth, this was a single-city study, which may limit the generalizability of our findings. Sixth, the “other locations” group included heterogeneous settings, which should be considered when interpreting comparisons across arrest locations.

## Conclusion

Initial shockable rhythm was identified in more than one-third of arrests occurring in nursing homes, and 97.0% of these cases received an AED shock. This may reflect earlier AED access and potentially increasing the likelihood of detecting a shockable rhythm. These findings underscore the importance of prompt AED use and suggest that extending AED access to home settings warrants further investigation.

## CRediT authorship contribution statement

**Chika Nishiyama:** Writing – review & editing, Writing – original draft, Visualization, Project administration, Methodology, Formal analysis, Data curation, Conceptualization. **Tomonari Shimamoto:** Writing – review & editing, Methodology. **Kosuke Kiyohara:** Writing – review & editing, Methodology, Conceptualization. **Tetsuhisa Kitamura:** Writing – review & editing, Methodology, Conceptualization. **Katsuya Tanaka:** Writing – review & editing, Resources, Investigation. **Tetsuya Sakamoto:** Writing – review & editing, Funding acquisition. **Taku Iwami:** Writing – review & editing, Supervision, Project administration, Methodology, Conceptualization.

## Funding

This study was supported by Grant-in-Aid for Health and Labour Sciences Research Grants (H29-Junkankitou-Ippan-009, 20FA1014, 23FA1023, 26FA1501) from the 10.13039/501100003478Japanese Ministry of Health, Labour and Welfare.

## Declaration of competing interest

CN is an editor of Resuscitation Plus. The rest of authors declare that they have no known competing financial interests or personal relationships that could have appeared to influence the work reported in this paper.
